# The Role of Nut and Seed Consumption in Colorectal Cancer: A Narrative Review

**DOI:** 10.3390/medicina58070932

**Published:** 2022-07-14

**Authors:** Deiana Roman, Bogdan Timar, Vlad Avram, Adina Braha, Sorin Saftescu, Șerban Negru, Romulus Timar

**Affiliations:** 1 Second Department of Internal Medicine, ″Victor Babeș” University of Medicine and Pharmacy, 300041 Timișoara, Romania; roman.deiana@umft.ro (D.R.); avram.vlad@umft.ro (V.A.); braha.adina@umft.ro (A.B.); timar.romulus@umft.ro (R.T.); 2 Department of Oncology, ″Victor Babeș” University of Medicine and Pharmacy, 300041 Timișoara, Romania; sorin.saftescu@umft.ro (S.S.); snegru@yahoo.com (Ș.N.); 3 OncoHelp Hospital, 300239 Timisoara, Romania

**Keywords:** colorectal cancer, dietary habits, nuts and seeds, nut consumption, clinical observations

## Abstract

Given the increased incidence of colorectal cancer worldwide, especially in developed and developing countries, is comes as no surprise that researchers are concentrating on methods to combat this public health issue, through investigating both lifestyle interventions and treatment options. Although treatment options are being constantly discovered and developed, researchers have also begun investigating the influence that nutrition and lifestyle have on CRC. Among the food categories, nuts and seeds boast numerous beneficial effects for cardiovascular health and metabolic balance and they contain a plethora of phytochemicals and antioxidants. The present narrative review aims to offer a broad perspective to date on the known effects of this consumption on colorectal cancer. For this purpose, articles were identified by conducting a search in the PubMed and Google Scholar databases, using search phrases such as ″nut intake and colorectal cancer″ and ″seed consumption and colorectal cancer”, narrowing the search pool to those articles published between 2019 and 2022. The search returned eight relevant papers, all of which were validated by a second author. While the existing research is divided between those studies which have found no significant link between nut consumption and colorectal cancer protection and those which have, there is a consensus regarding the necessity for further research on this subject, as well as the possible mechanisms which might be involved in the protective effect observed by some researchers.

## 1. Introduction

Cancer is ranked as one of the main causes of death worldwide, and the global cancer burden is expected to increase in the following decades [1]. Colon cancer is a leading unit in global cancer mortality in recent years, with approximately 10% of the number of cancer-related deaths being attributed to this disease [1,2]. Cancer prevention is of paramount importance in the containment of the global cancer burden [1].

Colorectal cancer incidence is among the highest in the world, with higher incidences being noted in countries transitioning towards higher economic status [1], implying that the socioeconomic shifts that occur during the development of a country have a profound influence on that society’s health, leading some to imply that colon cancer incidence is a sign of socioeconomic development [3].

Diet is an important factor that influences the incidence of colorectal cancer, with red meat consumption in particular being associated with a higher risk of colorectal cancer, while higher consumption of fruits and vegetables, as seen in the Mediterranean diet, seems to have protective effects [4,5]. Regarding treatment, it seems that lifestyle factors influence outcomes beyond the effects of chemotherapy and surgical interventions [6,7]. To date, increased patient BMI, a western type of dietary pattern and increased glycaemic load in patient diet have been associated with higher recurrence rates and increased mortality in stage III colorectal cancer, while physical exercise, a healthy Nordic diet or Mediterranean diet have shown protective effects [6,7,8,9].

The hallmarks of the Mediterranean diet are high fibre content derived from whole grains, protein and unsaturated fat, mostly based on fish-derived polyunsaturated fatty acids, associated with decreased content of saturated fats [10]. Furthermore, multiple servings of fruits and vegetables have also proven to be a beneficial attribute of this diet, with particular emphasis on the consumption of nuts [10].

Nuts have been a subject of increasing focus in regard to the improvement of overall health and mortality reduction in various diseases [11,12,13]. Apart from the well-known role that they have in reducing the incidence and mortality of cardiovascular diseases [10,14], there is an increasing body of evidence suggesting that nuts might aid in the prevention of cancer [15]. Nuts possess anticarcinogenic and antioxidant properties due to their content of tocopherols, phytosterols, folic acid and certain minerals, such as selenium or magnesium [16]. Polyphenols such as ellagitannins and urolithins, present in nuts, add to their anticancer properties, although the exact mechanisms of action are still under research and debate [17]. These compounds have been known to inhibit cancer cell proliferation, in colon cancer specifically, in a dose-dependent manner [18]. The phytochemicals found in nuts can behave in one of two ways, either inhibiting tumour initiation by acting as blocking agents (ellagic acid, flavonoids, indole-3-carbinol), or by halting tumour progression by acting as suppressing agents (betacarotene, resveratrol, inositol pentakiphosphate, inositol hexakisphosphate) [19]. Another possible mechanism that contributes to the benefits that nuts present in regard to colon cancer would be that nut consumption induces changes in the microbiota, which in turn modulate cell signalling and key cellular processes in the aspect of protein kinase signalling [17].

Although nuts in general are seen to have beneficial effects on health, there are differences in the magnitude or the effect itself that varies with each specific nut, which is not surprising seeing as nut composition varies within each nut type [11].

One of the earliest studies that included nut consumption in the recorded research items was ″The Nurses’ Health Study″ that was initiated in 1976 and that enrolled female nurses in the US, aged between 30 and 55 [20]. Published in 2016, the study prospectively followed approximately 76,000 women, and during the follow-up period, 1503 colorectal cancer cases were identified. At baseline, all participants had been cancer-free. In patients that reported a colorectal cancer diagnosis, permission to access medical records was requested [20].

Participants were asked to respond to a semiquantitative food frequency questionnaire. In 1980 and 1984, this questionnaire included inquiries regarding nut consumption over the preceding year: more than 6 servings a day, 4 to 6 servings a day, 2–3 servings a day, one serving a day, 5–6 servings per week, 2–4 servings per week, 1 to 3 servings per month or none/almost none. In the following questionnaires, there was a differentiation between peanuts and other nuts included, as well as a question dedicated to peanut butter consumption [20].

Consistency was maintained in regard to nut consumption throughout the study follow-up, those with higher nut consumption having a leaner body composition, being more likely to exercise, to take more multivitamin supplements, to consume more fruit and vegetables, as well as fibre, folate and calcium, being less likely to smoke, but more inclined to consume alcohol, as well as more likely to have lower endoscopy performed [20].

Consecutive to adjusting for other well-known or suspected risk factors for colorectal cancer, results showed that those female participants who consumed nuts with a frequency of two or more times per week, meaning more than or equal to 56 g of nuts per week, showed a decrease in colorectal cancer risk when compared to those with no or lower nut consumption, without reaching the threshold for statistical significance (RR: 0.87; 95% CI: 0.72–1.05; *P*_trend_: 0.06). For peanut butter in particular, there was no association to be observed in the studied group of participants [20].

The possible applicability and benefits of nut consumption in oncology have prompted research on this topic worldwide, and between 1990 and 2001, a prospective cohort study was conducted in seven townships in Taiwan and their respective precincts. In total, 12,026 men and 11,917 women with ages between 30 and 65 years were included in this prospective cohort study. Colorectal cancer cases were identified by means of data extraction from registries and death certificates [21].

Data regarding peanut consumption, as well as other dietary habits and food groups, were collected by means of structured questionnaire interviews administered by local nurses, who conducted home visits to all participants, evaluating various anthropometric measurements, socio-demographic parameters, family history of cancer and dietary and personal habits. Peanut consumption was evaluated as weekly frequency intake [21].

During the study, there were 107 confirmed new colorectal cancer cases, with 68 cases in male patients and 39 in women patients. Peanut consumption was associated with beneficial effects: RR = 0.83 (95% confidence interval (CI) = 0.44–1.21) in men and 0.75 (95% CI = 0.21–0.84) in women. A multivariate analysis using Cox’s proportional hazard model of the community-based cancer screening cohort revealed an RR of 0.73 (*p* = 0.22) in male participants and an RR of 0.42 (*p* = 0.01) in female participants, suggesting protective effects of peanut consumption against colorectal cancer in women, but not in men [21].

The European Prospective Investigation into Cancer and Nutrition (EPIC) published in 2004 included 10 European countries and was designed as a prospective cohort study. Within this larger body of research, a series of questions focused on the nut and seed intake of participants in relation to colorectal cancer risk [22].

The total nut and seed intake of participants was determined using a dietary questionnaire validated for all populations involved in the study. It is one of the largest, if not the largest, prospective cohort studies conducted investigating the link between diet and cancer [22].

The results of this study for the combined genders showed no notable protective association between nut and seed intake and the risk of colorectal cancer, whereas, upon subgroup analysis by participant gender, it was revealed that even an intake as modest as 16 g of nuts and seeds per day is associated with a reduced incidence of colorectal cancer (HR, 0.69; 95% CI, 0.50–0.95; fully adjusted model), particularly distal colon cancer (HR, 0.52; 95% CI, 0.32–0.85; fully adjusted model), in those women compared to those who did not consume nuts and seeds. This, however, does not apply to the male gender, though the reason for this disparity remains unclear and is to be further investigated [22].

Among studies focusing on the incidence of new cancer cases in regard to nut consumption in participants without cancer at baseline, there also are studies that have researched the impact that the consumption of nuts has on patients with diagnosed colon cancer, such as a study that included 826 patients with stage III colon cancer that completed food frequency questionnaires including 131 foodstuffs and vitamin and mineral supplements, while also leaving an open-ended section to be completed with other items that might not have been included in the questionnaire. Participants were questioned regarding the frequency of consumption of different items (in their respective portion sizes) over the previous three months, with nine possible answers, ranging from never to 6+ times per day. The median follow-up time was 6.5 years [23].

Those participants that consumed two or more servings of nuts per week experienced an adjusted hazard ratio (HR) for disease-free survival of 0.58 (95% CI, 0.37 to 0.92; *P*_trend_ = 0.03). If nut consumption was higher than previously mentioned, it was associated with a significant improvement in overall survival—HR of 0.43 (95% CI, 0.25 to 0.74; *P*_trend_ = 0.01). These results remained mainly unchanged, even after adjustment for confounding factors. Both disease-free survival and overall survival were improved significantly with a tree nut consumption increase, even though statistical significance could not be achieved regarding peanuts or peanut butter [23].

Among the limitations of this study, attention must be drawn to the self-reporting of nut intake, which could involve measurement errors and over- or underestimation of intake frequency or quantity. Dentition status was not among data collected within the cohort, although its poor condition might represent an impediment in the consumption of nuts, and dental disease has been associated with an increased incidence and mortality of cardiovascular disease [23].

While the existing research is divided between those studies which have found no significant link between nut consumption and colorectal cancer protection and those which have, there is a consensus regarding the necessity for further research on this subject, as well as the possible mechanisms which might be involved in the protective effect observed by some researchers.

Researchers have identified four major phenolic compounds in the walnut phenolic extract (WPE), namely gallic acid, catechin, chlorogenic acid and ellagic acid in various quantities, catechin being the most abundant, followed by chlorogenic acid, ellagic acids and, lastly, gallic acid [24].

CD133^+^, CD44^+^, and HCT116 cells were treated with WPE for 2,4 and 6 days, showing a suppression of cell growth in a manner that was dose-dependent. In particular, 40 μg/mL WPE was shown to inhibit cell growth by up to 34.4% (*p* < 0.01) after 2 days, 59.1% (*p* < 0.001) after 4 days and 85.8% (*p* < 0.01) after 6 days when compared to control cells. Consecutively, it was determined that WPE extract showed the highest efficacy at 4 and 6 days, while its individual bioactive compounds were not found to have significantly different effects on cell growth after 4 and 6 days of treatment [24].

mRNA levels of established cancer stem cell (CSC) markers including CD133, CD44, DLK1 and Notch1 underwent RT-PCR investigation, revealing suppression of their expression by WPE in a manner that was dose-dependent. Furthermore, comparable dosages of the bioactive compounds equivalent to 40 μg/mL WPE also proved highly effective in the suppression of the four CSC markers. By comparison, though, WPE was found to be superior to the individual bioactive compounds in regard to the down-regulation of the expression of these CRC markers [24].

WPE was also shown to suppress the self-renewal capacity of colon CSC through the observation of single cells and their ability to form a colony. When CD133^+^, CD44^+^ and HCT116 cells underwent treatment with WPE in various concentrations, the number of cells that were able to form colonies showed a decrease in a dose-dependent manner, up to 94%. Again, WPE as a whole compared to each individual bioactive component showed superior efficacy in the colony formation suppression [24].

Sphere formation has also been shown to decrease in a dose-dependent fashion, cells treated with WPE having an inferior capacity to form spheres, this suppression rising to 72.3%, compared to non-treated cells, indicating that WPE as well as its bioactive compounds are able to suppress CSC by regulating self-renewal capacity [24].

In order to confirm the applicability of these findings in the human CRC tissue, primary cells from this tissue were isolated and received treatment with WPE in varying doses, with results showing a significant down-regulation in mRNA levels of CD133^+^ markers by 62%, of CD44^+^ markers by 33.5%, of DLK1 markers by 57.1% and of Notch1 markers by 81.1% compared to control cells [24].

Among the chief characteristics of telomerases is their ubiquitous presence in nearly all cancer cells, and lack thereof in most normal cells. Given the fact that telomere maintenance is of the utmost importance in cancer proliferation, the inhibition of telomere maintenance could prove to be a significantly useful approach in cancer treatment. Cancer cell death could, thus, be induced through the targeting of the telomerase and telomere connection [24].

Another study focused on walnuts (*Juglans regia* L.), given their anti-inflammatory and antioxidant properties, alongside their cardioprotective ones. In order to specifically determine the mechanism through which walnuts exert their inhibitory effect on the growth of cancer cells, telomere activity, as well as length, was determined in a stem cell model. In cells treated with walnut phenolic extract (WPE), telomere length had significantly decreased in a dose-dependent manner (5.16 ± 0.13 at 0 µg/mL, 4.79 ± 0.12 at 10 µg/mL, 3.24 ± 0.08 at 20 µg/mL, and 3.99 ± 0.09 at 40 µg/mL; *p* = 0.0276). Telomerase activity was also observed to decrease proportionally with WPE concentration (1.47 ± 0.04, 1.09 ± 0.01, 0.76 ± 0.08, and 0.88 ± 0.06; *p* = 0.0067). Transcriptions of hTERT and c-MYC were also observed to significantly decrease in a stepwise pattern, proportionally with the stepwise increase in WPE concentration. The mechanisms underlined by this study might represent those through which walnuts inhibit cancer cell growth [25].

In a case–control study conducted in Korea, researchers wanted to investigate the link between nut consumption and the risk of colon cancer, their study being published in 2018 in the Nutrition Journal. A total of 923 colon cancer patients were included, alongside 1846 controls. Dietary habits were collected through a semi-quantitative food intake questionnaire. The questionnaire included 106 food items, among which were almonds, pine nuts and peanuts. Regarding frequency of consumption, there were three categories: less than once a week, between one and three servings per week and three or more servings per week [26].

A significant association was observed between high nut consumption and a reduction in colorectal cancer among women (in those with an intake of ≥ 3 servings of nuts per week compared to those that declared no nut consumption). A similarity in association was also observed in the male gender for those with a nut consumption of ≥ 3 servings per week compared to those with none. When researchers performed sub-site analyses in men comparing the high intake group to the no intake group, adjusted OR ratios were 0.25 (95% CI: 0.09–0.70) for proximal colon cancer, 0.39 (95% CI: 0.19–0.80) for distal colon cancer and 0.23 (95% CI: 0.12–0.46) for rectal cancer. In women, the same sub-site analysis found an inverse association for distal colon cancer (OR = 0.13, 95% CI: 0.04–0.48) and for rectal cancer (OR = 0.40, 95% CI: 0.17–0.95) [26].

In conclusion, the findings of the present study come in support of the important role that nut consumption plays in the prevention of colon cancer, in both men and women, in all sub-sites of the colon, except for the proximal colon in women [26].

The Netherlands Cohort study, conducted over the course of 20.3 years, found no significant association between nut intake and colorectal cancer in categorical and continuous analyses. In women, in restricted cubic spline analyses, a significant inverse association was observed regarding the risk for rectal cancer and peanut, peanut butter and nut consumption, while non-linearity analysis showed significance only for nut and peanut consumption, not for peanut butter. In the case of the male gender, only borderline significance was achieved upon examination of the inverse non-linear relation between nut or peanut consumption and rectal cancer risk [27].

Fruit, vegetables and nuts are rich in campesterol, β-sitosterol and stigmasterol. These phytosterols have been linked to colon cancer risk reduction in past studies. It is perhaps through their high concentration in these substances that nuts reduce colon cancer risk [28,29].

An inverse association was found between consumption of phytosterols and cancer risk. Among the 11 performed comparisons, intense heterogeneity was present (*p* < 0.001), finding a pooled RR of 0.63 (95% CI = 0.49–0.81) for the highest versus the lowest intake of phytosterols. Five comparisons were evaluated in two articles and found a pooled RR of 1.12 (95% CI = 0.96–1.32) for cancer risk with consumption of β-sitostanol, with no evidence of heterogeneity. The highest versus the lowest intake of campestanol and cancer risk was evaluated in two articles with five comparisons, finding a pooled RR of 0.82 (95% CI = 0.63–1.06) for cancer risk, with little evidence of heterogeneity [30]. The mechanisms through which phytosterols influence cancer development and progression have been conveniently summarised in Table 1 [31].

Phytosterols have cholesterol-lowering effects that have beneficial anticarcinogenic effects beyond the well-known cardiovascular benefits [32]. Lower cholesterol levels have proven beneficial in lowering cancer progression, migration and invasion, modifying various tumor-generating pathways such as modifying VEGF expression, influencing LDL-receptors, decreasing the expression of Niemann–Pick C1-like 1 transporter and reducing reactive oxygen species [32,33]. There is also evidence of the contribution of phytosterols to reducing cancer risk by means of their anti-inflammatory properties, such as decreasing nuclear translocation of nuclear factor kappa B [32]. Further anticancer properties of phytosterols may be through their activation of AMP-kinase, an enzyme that is also activated by metformin, which has been long known to have beneficial effects regarding cancer [32,34]. Moreover, they have been found to increase interleukin 2 and interferon-γ, possibly limiting tumor metastasis [32].

Table 2 provides a summarization of the articles included in the present review, alongside their main conclusions.

## 2. Conclusions

Nut and seed consumption has shown multiple benefits for the health of individuals; nowadays, the scientific community poses an intriguing and important question regarding its effect in cancer, particularly colorectal cancer. In the attempt to answer this question, multiple studies have been conducted, leading to often contradicting results. The study design, selected population, patient clinical status, self-declared nut intake, memory bias and a host of other inconsistencies might be at the root of these discrepancies. Knowledge to date infers possible mechanisms through which nuts and seeds could influence the development and progression of colorectal cancer, underlining the need for further research in this particular domain.

## Figures and Tables

**Table 1:** Phytosterols and anticancer mechanisms.

Main Phytosterols	Anticancer Mechanisms
Campesterol β-Sitosterol Stigmasterol Sitostanol Sigmastane Campemastane Cholesterol	Alteration of membrane fluidity Alteration of membrane integrity Alteration of membrane-bound enzymes Alteration of signal transduction pathways apoptosis Immune function Tissue estrogenic properties Neutral and acidic sterols in the colon, anti-inflammatory effects

**Table 2:** Summary of study results.

Study	Results	References
The Nurses’ Health study Peanut consumption and reduced risk of colorectal cancer in women: a prospective study in Taiwan.	Decreased risk of colorectal cancer (CRC) for those who consumed 2+ servings of nuts per weekSuggests protective effects of peanut consumption against CRC in women	[20]
		[21]
The European Prospective Investigation into Cancer and Nutrition (EPIC)Nut Consumption and Survival in Patients With Stage III Colon Cancer: Results From CALGB 89803 (Alliance)	Even an intake of 16 g of nuts and seeds per day reduced incidence of CRC in women	[22]
	Both disease-free survival and overall survival in stage III CRC patients were significantly improved with tree nut consumption increase	[23]
Walnut Phenolic Extract and Its Bioactive Compounds Suppress Colon Cancer Cell Growth by Regulating Colon Cancer Stemness		
	Suppression of cell growth in a dose-dependent manner Inhibition of telomere maintenance	[24]
Walnut phenolic extracts reduce telomere length and telomerase activity in a colon cancer stem cell model		
	In cells treated with WPE, telomere length had significantly decreased in a dose-dependent manner Telomerase activity was also observed to decrease proportionally with WPE concentration	[25]
The relationship between nut intake and risk of colorectal cancer: a case control study		
	Underlines the important role that nut consumption plays in the prevention of colon cancer, in both men and women, in all sub-sites of the colon	[26]
Nut and peanut butter intake and the risk of colorectal cancer and its anatomical and molecular subtypes: the Netherlands Cohort Study		
	In women, in restricted cubic spline analyses, a significant inverse association was observed regarding the risk for rectal cancer and peanut, peanut butter and nut consumption In the case of the male gender, only borderline significance was achieved upon examination of the inverse non-linear relation between nut or peanut consumption and rectal cancer risk	[27]

## Data Availability

Not applicable.
